# In-situ STEM imaging of growth and phase change of individual CuAl_X_ precipitates in Al alloy

**DOI:** 10.1038/s41598-017-02081-9

**Published:** 2017-05-19

**Authors:** Chunhui Liu, Sairam K. Malladi, Qiang Xu, Jianghua Chen, Frans D. Tichelaar, Xiaodong Zhuge, Henny W. Zandbergen

**Affiliations:** 1 Kavli Institute of Nanoscience, Delft University of Technology, 2628 CJ Delft, The Netherlands; 2 Center for High Resolution Electron Microscopy, College of Materials Science and Engineering, Hunan University, 410082 Changsha, China; 3 DENSsolutions, Informaticalaan 12, 2628 ZD Delft, The Netherlands; 4 Computational Imaging (CI), Centrum Wiskunde & Informatica (CWI), Science Park 123, 1098 XG Amsterdam, The Netherlands; 5 Department of Materials Science and Metallurgical Engineering, Indian Institute of Technology Hyderabad, Kandi, Sangareddy 502285 Telangana, India

## Abstract

Age-hardening in Al alloys has been used for over a century to improve its mechanical properties. However, the lack of direct observation limits our understanding of the dynamic nature of the evolution of nanoprecipitates during age-hardening. Using *in-situ* (scanning) transmission electron microscopy (S/TEM) while heating an Al-Cu alloy, we were able to follow the growth of individual nanoprecipitates at atomic scale. The heat treatments carried out at 140, 160, 180 and 200 °C reveal a temperature dependence on the kinetics of precipitation and three kinds of interactions of nano-precipitates. These are precipitate-matrix, precipitate-dislocation, and precipitate-precipitate interactions. The diffusion of Cu and Al during these interactions, results in diffusion-controlled individual precipitate growth, an accelerated growth when interactions with dislocations occur and a size dependent precipitate-precipitate interaction: growth and shrinkage. Precipitates can grow and shrink at opposite ends at the same time resulting in an effective displacement. Furthermore, the evolution of the crystal structure within an individual nanoprecipiate, specifically the mechanism of formation of the strengthening phase, θ′, during heat-treatment is elucidated by following the same precipitate through its intermediate stages for the first time using *in-situ* S/TEM studies.

## Introduction

Age hardening in aluminium alloys, which involves the growth of extremely fine structures ranging from a few atomic layers to precipitates as large as a few hundred nanometres, is a process over hundred years old^1, 2^, and has been successfully applied on an industrial scale to strengthen light-weight metal alloys^3, 4^. During age-hardening, a metal alloy is heated to an elevated temperature to form a completely homogeneous solid-solution and then rapidly cooled to room temperature (*quenching*) to form a super-saturated solid-solution. From this condition, upon heating to temperatures higher than room temperature (typically around 100–200 °C for Al alloys, known as *thermal ageing*), the alloy decomposes resulting in the formation of a dispersion of nanoprecipitates in the matrix by the segregation of the alloy’s solute atoms. These nanoprecipitates enhance the strength of an alloy by impeding the movement of dislocations when subjected to an external force. Extensive experimental^5–8^ and theoretical^9–17^ research has been carried out to understand the underlying mechanism of the morphological evolution of the precipitates in Al alloys. In general, it is thought that the nanoprecipitates grow by diffusion of solute atoms towards the nucleation sites. They coarsen via the diffusion of individual atoms from neighbouring precipitates following the Gibbs-Thomson effect^18^. Some theoretical simulations^13–16^ predict that precipitates with a diameter of a few nanometres can coalesce by the displacement of a precipitate as a whole. Until now, such coalescence has not been observed experimentally. Our understanding on precipitate sequences is based on conclusions drawn from high resolution analytical techniques like TEM and 3D-atom probe tomography (3DAPT) carried out on different samples *post mortem* (after heat-treatment). Owing to statistical differences from of a range of samples, it is hard to establish a link between the evolution of the same individual precipitate at different stages of evolution. To determine the evolution of single precipitates and establish a link between the theoretical predictions and the intermediate stages of precipitation, we have performed TEM studies while heating *in-situ*.

A high-purity binary alloy, Al-Cu 5.7 wt. % (with impurities such as Mg, Si < 0.02 wt%), chosen for this work possesses a strong age-hardening potential and is widely used as a commercial Al alloy (see Supplementary Information Fig. S1 for more details). It is experimentally demonstrated that the most effective strengthening phase in Al-Cu alloys is the plate-like θ′ precipitate (with a space group of I4/m, a = 0.404 nm, c = 0.580 nm)^19^. Prior to the formation of the θ′ phase, the {100} plate-like precipitates undergo a complex evolution in atomic structure and chemistry^9, 10, 20^. A detailed knowledge of this evolution is required to reveal the formation mechanism of the strengthening precipitates, as this can be used for optimising heat treatments in the production process.

We have studied the evolution of individual precipitates and their interactions using *in-situ* STEM. For this we used the micro-electro-mechanical systems (MEMS) based heating holder^21, 22^ which allows precise control of the temperature, a very low specimen drift and high stability of the sample during heating. This allowed keeping the sample area in view during the whole ageing process, which was in some cases as long as 20 h. A lamellar specimen, prepared by a focused ion beam, with a thickness of 230–250 nm was placed on the MEMS heater (see Methods and Supplementary Information section 1 & 2 for more details). Annular dark-field STEM (ADF-STEM) at the atomic-scale was performed on a C_S_-corrected FEI Titan TEM while heating the specimen *in-situ*. First we heated the specimen using the MEMS heater to a temperature of 520 °C to form a single-phase solid solution (*solutionising*) and then switched the MEMS heater off for spontaneous cooling to the ambient temperature (*quenching*). Subsequently, the specimen was heated at various temperatures in the range of 140–200 °C for several minutes to several hours. This heating cycle resembles the thermal history of an industrial ageing treatment. Importantly, the heat treatment can be repeated on the same lamella and the results are reproducible.

## Results and Discussion

### Real-time observation of the growth of precipitates in the Al-Cu alloy

**Figure 1:**
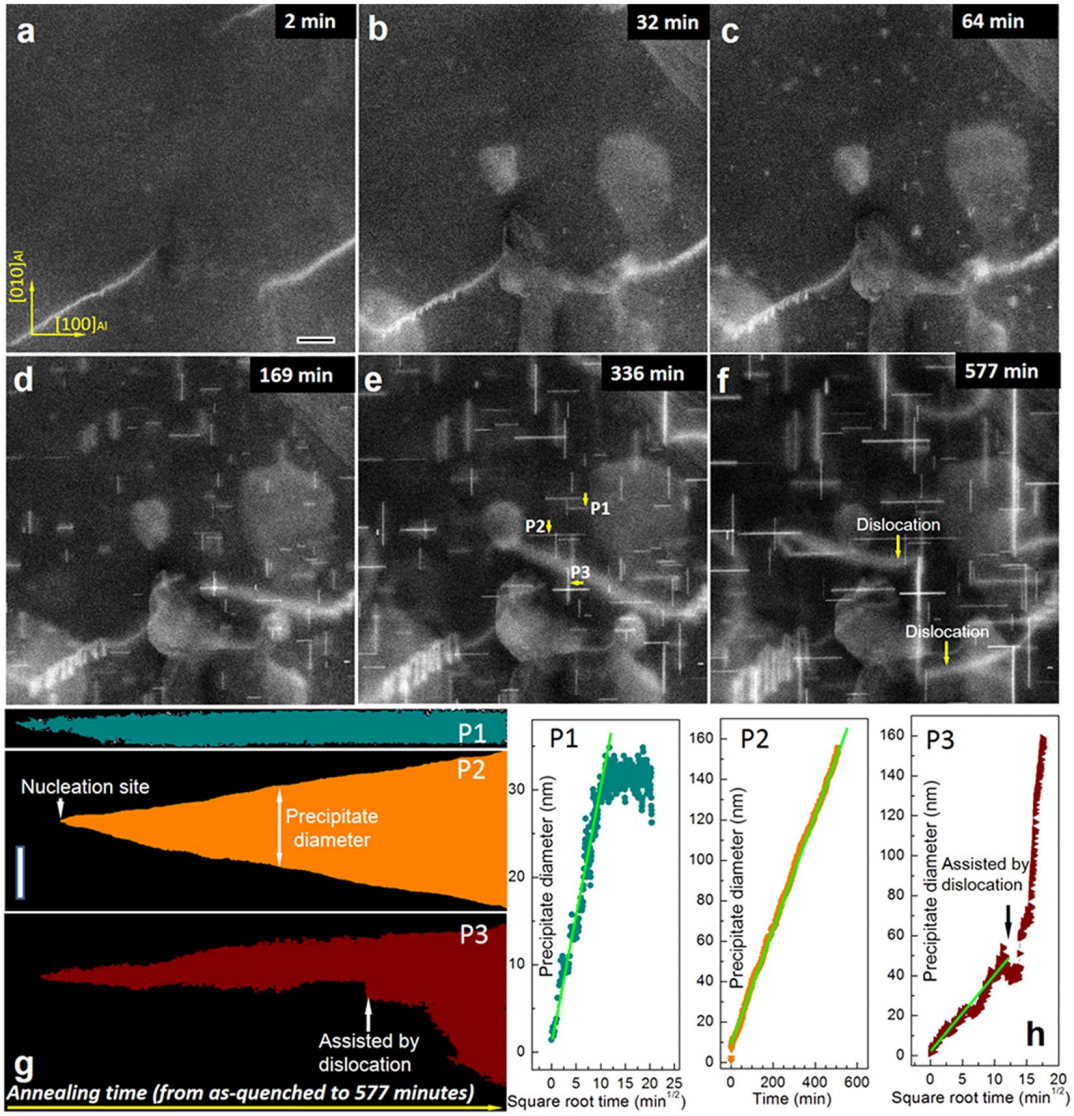
Real-time observation of the growth of precipitates in Al-Cu alloy. (**a**–**f**) Snapshots from an *in-situ* ADF-STEM Movie S2 showing the growth of plate-like precipitates in the Al-Cu alloy during ageing at 160 °C, with the electron beam parallel along the 〈001〉_Al_ direction. The small sub 5 nm bright spots increasing in contrast in (**a**,**b**,**c**) are small precipitates growing into the larger precipitates visible as lines in (**d**,**e**,**f**). The larger (up to 150 nm diameter) bright areas are copper enriched precipitates formed at the foil surface. (**g**) Average length diameter of seven precipitates monitored from the moment of nucleation. (**h**) The growth trajectories of the precipitates P1, P2 and P3, formed at various stages of ageing. The horizontal scale bar in (**a**) is 50 nm and refers to all figures (**a**–**f**). The vertical scale bar in (**g**) is 50 nm.

We recorded several time-series of low-resolution ADF-STEM movies during ageing at various temperatures. Although such images do not resolve the atomic structure, they are essential to obtain a good overview of the precipitation processes and provide statistical information. In ADF-STEM imaging mode the intensity in every pixel is approximately proportional to *Z* ^1.5~1.8^ (*Z* is atomic number)^23, 24^ and thus a bright pixel reflects the presence of Cu. The Movies S1–S4 (see Supplementary Information for more details) show the evolution of precipitates at 140, 160, 180 and 200 °C respectively. Figure 1 shows a sequence of images from Movie S2, revealing the precipitate morphologies at different stages of ageing at 160 °C. At first, one can observe bright areas (1–2 nm large) that can also disappear, which we interpret as Cu rich compositional fluctuations. Some of these bright areas develop at a later stage into plate-shaped precipitates. The Movies S1–S4 show that upon increasing the ageing temperature: (a) the nucleation rate is higher, (b) the precipitate growth-rate is larger, and (c) the precipitate density is lower. The growth of individual precipitates was determined from the movies. On comparing the Movies S1–S4, we find that (i) the widths of the precipitates are the same, (ii) the growth of a precipitate is stopped when it meets another perpendicularly oriented precipitate and (iii) the growth of a precipitate is strongly enhanced when it coincides with a dislocation. Two effects occur only at 140 and 160 °C: the presence of Cu-rich areas (see 50–150 nm bright cloud-like regions surrounding the precipitates in Fig. 1c and d, Movie S1 and S2); and the “merging” of nearby precipitates (see Movie S1 and S2). The theories on diffusion-limited precipitate growth in Al alloys predict that the length of the precipitate ‘*L*’ varies with time of heat treatment ‘*t*’ as *L* ∝ *t* ^*n*^, where n = 1/2 for diffusion-controlled growth and n = 1/3 for Ostwald ripening^23, 24^. In this *in-situ* S/TEM study, the projected precipitate lengths can be measured, given that (i) the thickness for the precipitate plates remains almost unchanged during the entire ageing, (ii) we image the plates edge-on and (iii) we can assume that the plates are elongated discs. From Fig. 1d–f, it is evident that there is no significant change in the precipitate width. As our experimental data is acquired by orienting the Al specimen precisely along the (001)_Al_ axis, conditions (i) and (ii) are satisfied. For condition (iii), S/TEM tomography measurements after the *in-situ* heat-treatment confirm that the precipitate plates are like elongated discs (see section 7 in Supplementary Information). Note that our experimental data differs in two aspects from post-mortem TEM analysis of a series of bulk samples: (i) when precipitates grow up to the sample surface their growth behaviour will change, and (ii) the precipitates that grow fast due to the presence of a dislocation can be identified and treated differently in the statistical analysis, which is possible only with *in-situ* TEM.

The growth trajectories of precipitates (Fig. 1g) and variation of the precipitate length (Fig. 1h) were extracted from twenty random precipitates from each of the frames in the Movies S1–S4. Three of the representative precipitates are shown here in Fig. 1g (see sections 2–4 of Supplementary Information for details). A significant fraction of precipitates (17 out of 20) show volume-diffusion-controlled growth like P1 (‘*n*’ = ½ see Fig. 1h) till they reach a specific length and are limited by either the alloying element or the thickness of the specimen. A small fraction of precipitates (3 out of 20) show a much faster growth like P2 (‘*n*’ ≈ 1), indicating a much faster growth rate that could be surface assisted. There are a few exceptional cases like P3, which follow a volume diffusion controlled growth till connecting to a dislocation, resulting in an accelerated growth. (see Supplementary Information part 3 and part 4 for more details). At higher ageing temperatures of 180 °C and 200 °C (see Supplementary Movies S3 and S4), the growth behaviour of the precipitates is quite similar to those at lower temperatures of 140 °C and 160 °C, except for the growth speed. A larger fraction of the precipitates changes from a volume-diffusion controlled growth to a more linear growth, which is likely due to the effect of the surface as the precipitates grow large enough to extend to the surface of the sample (also see Supplementary Information part 3).

### Nucleation and growth of an initial-stage precipitate

**Figure 2:**
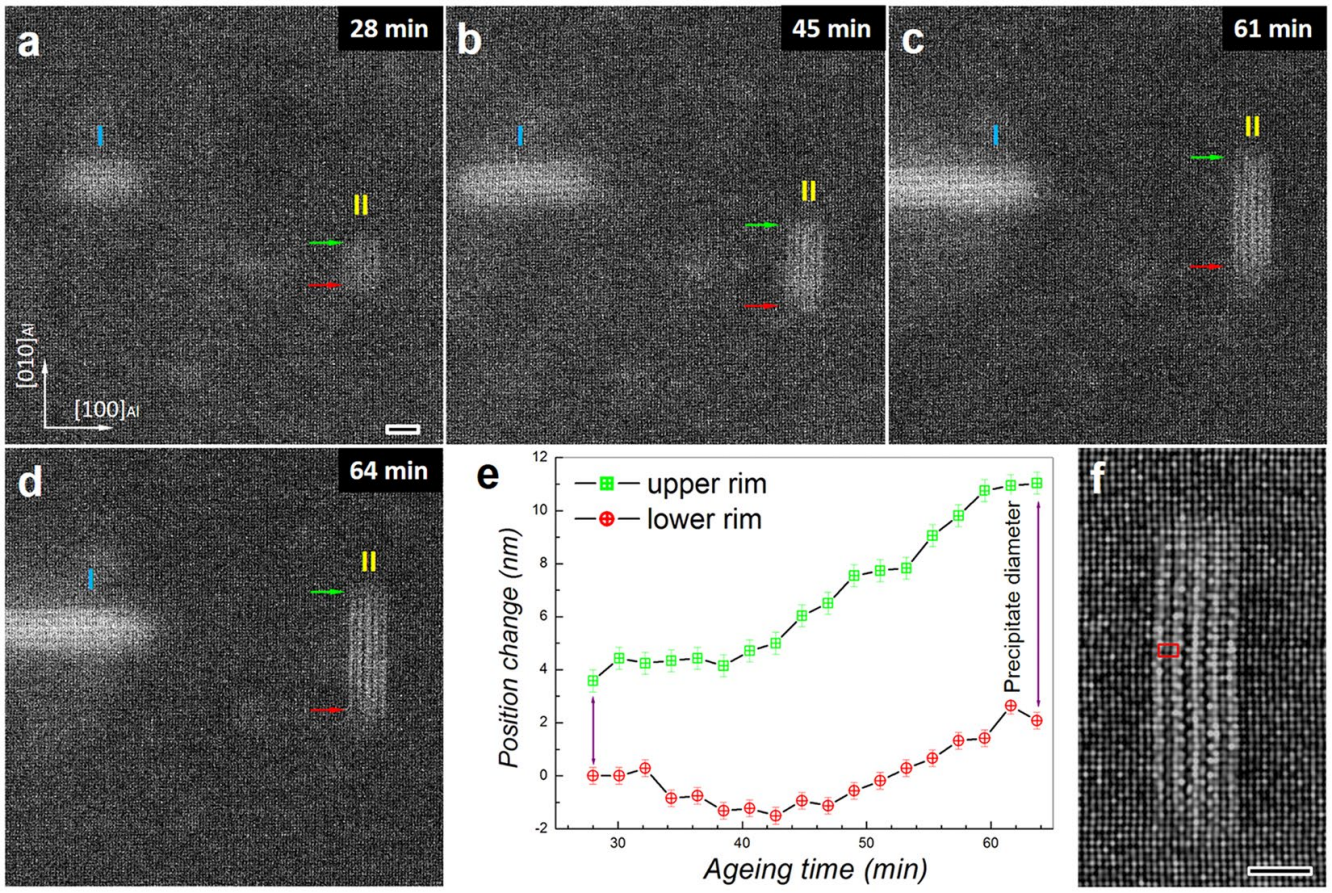
The growth of an initial-stage precipitate. Snapshots taken from HR-STEM Movie S5 obtained after heating the quenched Al-Cu alloy at 160 °C for (**a**) 28 min, (**b**) 45 min, (**c**) 61 min, and (**d**) 64 min. The blue arrow and red arrow point to the upper rim and lower rim, respectively. (**e**) The change of the positions of upper rim and lower rim of this precipitate. (**f**) The atomic-scale ADF-STEM image of the precipitate in (**d**). Scale bars (**a**–**d**) 2 nm, (**f**) 2 nm.

**Figure 3:**
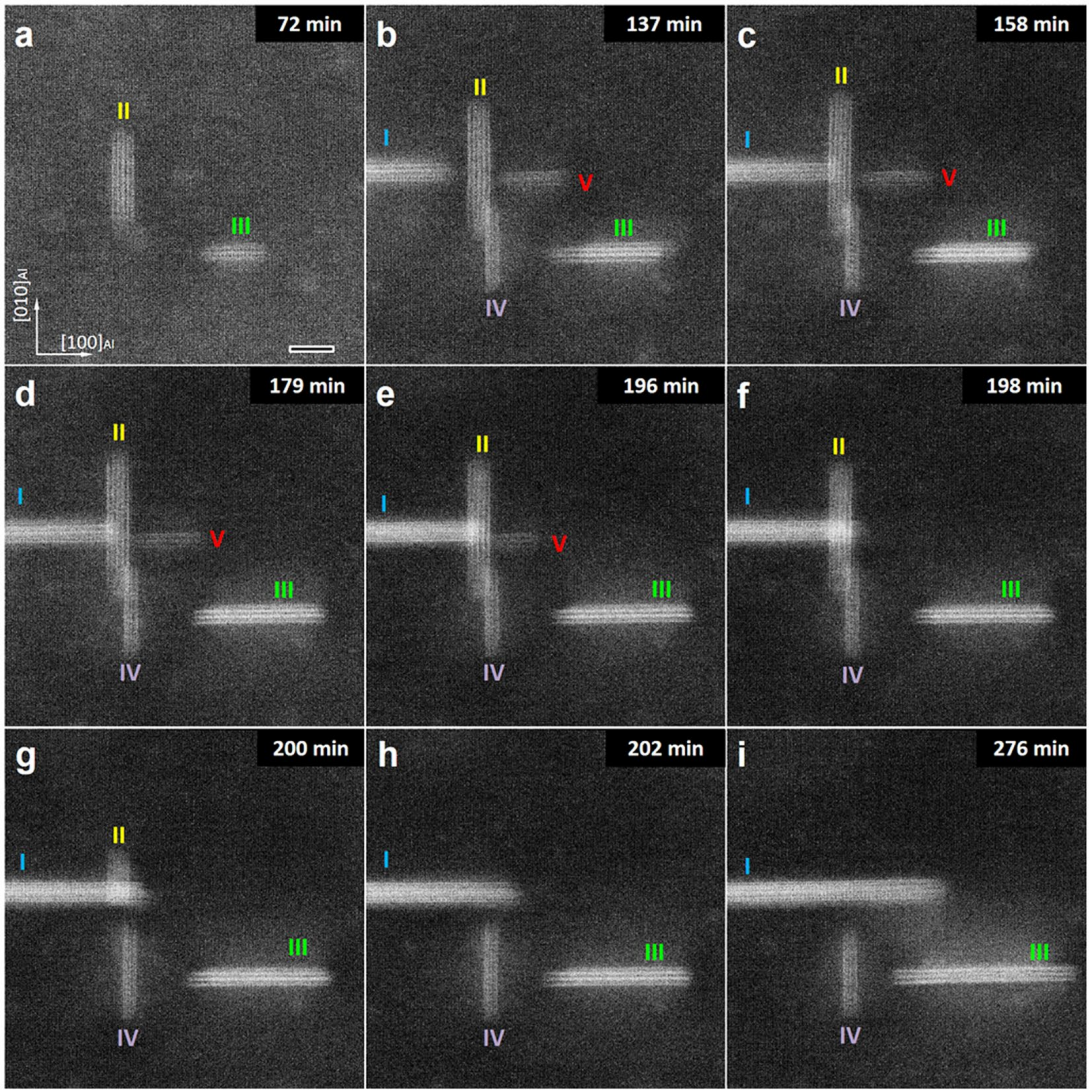
The interaction of precipitates in close proximity to each other. Snapshots taken from HR-STEM Movie S5, showing the features of precipitate morphology at various moments during the heat-treatment of the Al-Cu alloy at 160 °C. It is evident that precipitate I grows further and faster after coming in contact with precipitates II and V, by absorbing them. Scale bars are 5 nm in all images.

We recorded high-resolution movies during ageing at 160 °C to obtain atomic scale information on the nucleation and growth of nanoprecipitates (see Movie S5). Snapshots from this movie are shown in Fig. 2 (images from 20–64 min) and Fig. 3 (from 72–276 min). In the first 20 min, some brighter areas appear which we attribute to aggregates of Cu. Two of the five aggregates develop into precipitates. Several stages of the growth of these two precipitates are observed as shown in Fig. 2. Precipitate I (P-I) grows faster than precipitate II (P-II). Since both precipitates are perpendicular to each other and do not move along their plate normal, the growth or shrinkage of both rims of the precipitates can be determined precisely. The graph in Fig. 2e displays the positions of the upper rim and lower rim of P-II, using a horizontal line from the centre of P-I as a reference. The change in positions of the two rims of P-II is quite remarkable; after approximately 45 min ageing the precipitate extends in one direction and retracts in the opposite direction and is thus displacing (apart from growing). It implies that precipitate growth by diffusion of Cu to the upper rim is competing with dissolution of Cu at the lower rim. Using P-II as reference for position, one can see that P-I is growing faster on the side facing away from P-II while there is no growth at all on the side facing P-II.

### Interaction of precipitates during growth

Figure 3 shows the precipitates P-I and P-II from Fig. 2 as well as three other precipitates, which were not yet formed during the first 64 minutes of ageing. Prolonged ageing at the same temperature for 2 hours results in the formation of a new precipitate (P-III in Fig. 3a) followed by two more precipitates (P-IV and P-V in Fig. 3b). After 158 min of ageing, it appears that P-I is in contact with P-II in the projected STEM image (see the 3-D illustration in Fig. S7f). Since the lateral growth of P-I is halted from 158 min to 196 min, we conclude that this is indeed the case in 3D. This observation is supported by the continued growth of P-I along the viewing direction, which is evident from the increase in brightness. For the following 20 min, there is little change, except for a brightening of P-I and a slight shrinking of P-II and P-V. From 196 to 202 minutes, P-V and P-II vanish. It is striking that the relatively large P-II is dissolved in 4 minutes. In the following 80 minutes P-I and P-III grow continuously whereas P-IV does not change significantly (Fig. 3h and i). During this growth, a bright cloud surrounding P-III due to the solute (Cu) enrichment is observed. The whole process can be seen in Movie S5 and the corresponding analysis is detailed in part 5 of Supplementary Information.

### Atomic structure evolution of an individual precipitate

**Figure 4:**
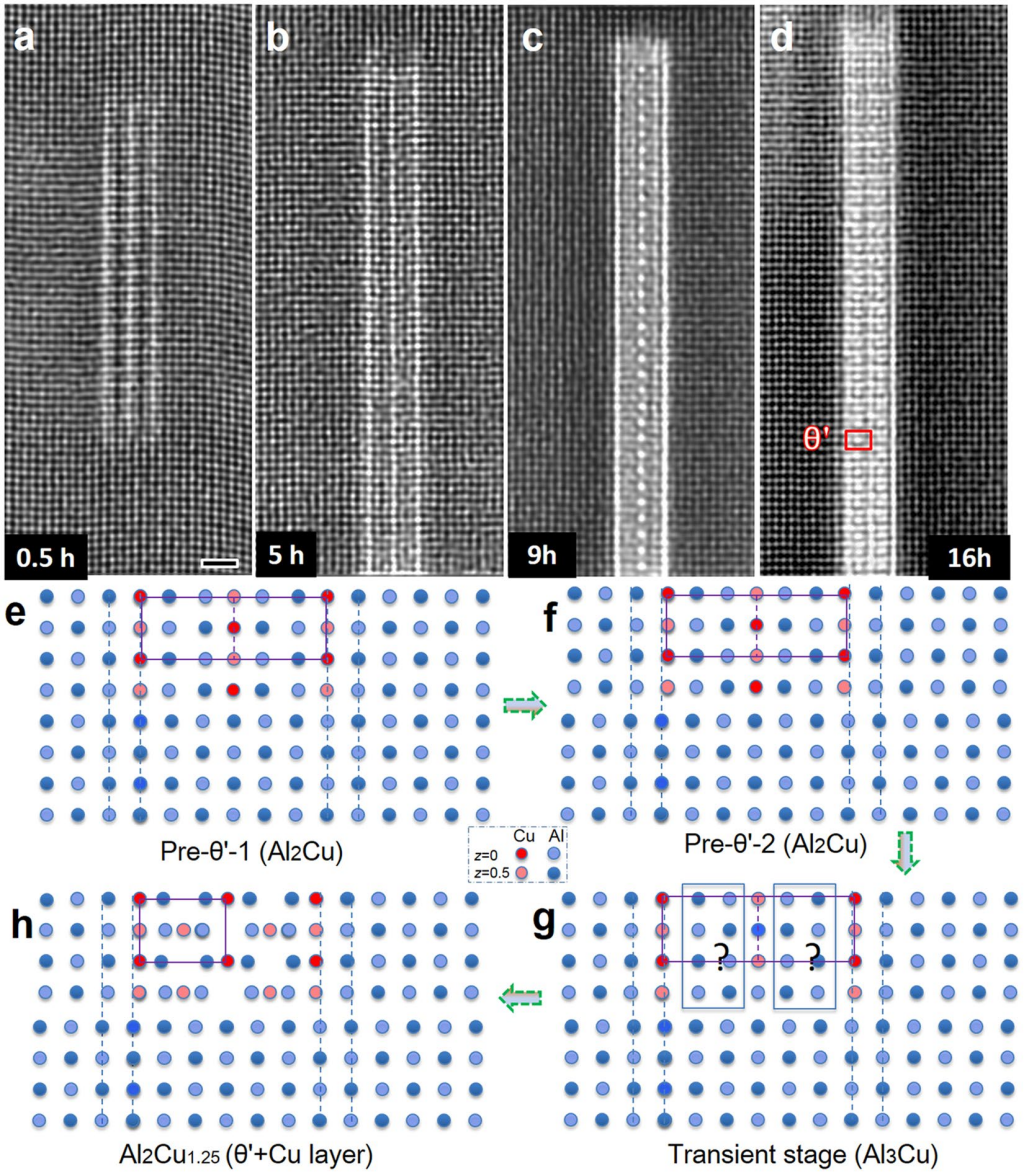
Atomic structure evolution of an individual precipitate. (**a**–**d**) The HR-STEM images showing the atomic structures of the same precipitate in Al-Cu alloy during ageing at 160 °C for 0.5 h (**a**), 5 h (**b**), 9 h (**c**) and 16 h (**d**). The STEM images are Fourier filtered to reduce the noise. (**e**–**g**) Structural models of the various phases of the phase transformation occurring inside this precipitate. In order to distinguish the lattice expansion/contraction in the intermediate stages, dotted lines are shown next to the precipitate and the lattice contraction/expansion is strongly increased for illustration purposes. Scale bars (**a**–**d**), 1 nm.

Figure 4 shows atomic-resolution ADF-STEM images of the same precipitate at various growth stages while aging at 160 °C. With prolonged ageing time, the precipitate’s plate diameter increases, but keeps the same thickness, indicating that the outer Cu layers’ act as a stable skeleton during the ageing process. The structure inside the precipitate evolves gradually from the pre-θ′-1 (Fig. 4a), through the intermediate phase pre-θ′-2 (Fig. 4b) and a transient stage (Fig. 4c) to θ′ (Fig. 4d), whereby the internal atomic configuration in θ′ changes strongly. Schematic models for pre-θ′-1, pre-θ′-2 and θ′ are shown in Fig. 4e–h. Line scans along the horizontal direction show the grey level variation in the precipitate, indicative of the amount of Al to Cu replacement (see part 6 of Supplementary Information and Fig. S11 for the line scans). Using these line scans, the compression/expansion of the lattice between the two outer Cu planes was determined as: no lattice change for Fig. 4a; a compression of 0.2 Å for Fig. 4b; an expansion of 0.1–0.2 Å for Fig. 4c and a compression of 0.1–0.2 Å for Fig. 4d respectively. Note that since the precipitate is embedded in the matrix along the viewing direction, the obtained STEM image has an overlap of the Al matrix and precipitate, resulting in a less clear image of the precipitate. Likewise, the STEM image taken close to the precipitate will contain an overlap of undistorted Al and the distorted Al close to the precipitate, resulting in a blurred image. This image blurring (for instance in Fig. 4b–d and not in Fig. 4a) of the surrounding Al matrix is a sign for lattice displacement.

Using the precipitate structures deduced from Fig. 4, DFT calculations were carried out to estimate the optimized structure parameters and formation enthalpy of the intermediate stages. These observations can be explained as: Pre-θ′-1 (Al_2_Cu) consists of three Cu layers separated by two Al layers - the displacement of two Al atom layers towards the Cu layers results in a dark contrast in the STEM image (Fig. 4a,e) - thus there is relatively more space (~50 pm) between these Al layers resulting in a path of easy diffusion for Al and Cu atoms (of 143 pm and 128 pm diameters respectively). In pre-θ′-1, the positions of the outer Cu layers (Fig. 4e) are the same as those of the Al lattice because a contraction is energetically unfavourable for this small precipitate. When the precipitate is large enough to overcome the strain energy of the matrix, the precipitate lattice contracts as observed for pre-θ′-2 (Fig. 4b,f). In the next stage (Fig. 4g), the precipitate is expanded with respect to the Al lattice (see part 6 of Supplementary Information for details). We attribute this expansion to atomic rearrangements related to the (local) transformation of the Al based lattice of the pre-θ′-2 phase. In this transient phase, the Cu plane in the middle is composed of alternating bright and less bright dots indicating a Cu-Al alternation. Between the middle Cu plane and the two Cu planes on the edges of the precipitate, we do not observe a clear, well-defined structure, which we attribute to a variation due to a transformation along the viewing direction. Subsequently the atoms in between the Cu planes undergo an ordering process and finally the complete structure of θ′ is formed (Fig. 4h). DFT calculations (see part 6 of Supplementary Information) indicate that the pre-θ′-1 phase prefers to contract, resulting in the phase change to the pre-θ′-2 phase. Likewise, θ′ shows a contraction along the Cu plate normal. We have tried various models for the transient phase (Fig. 4c) between pre-θ′-2 and θ′ and they all show a similar contraction. All these models assume a fully ordered structure over the whole thickness of the precipitate, whereas we believe that that is not the case in our experiments and that the shift of the Cu atoms to non-Al lattice sites requires overcoming an energy barrier. By estimating the formation enthalpies of intermediate precipitate structures, the most favourable sequence for the formation of θ′ precipitates is determined by DFT calculations as detailed in part 6 of Supplementary Information. This kinetic pathway is consistent with the observed formation mechanism of θ′ precipitates. The coherent interfaces of θ′ with aluminium recently found to be continuous Cu-rich layers^25^ are similar to those shown in Fig. 4d.

The very fast disappearance of the two precipitates P-II and P-IV in Movie S5 and Fig. 3 requires a fast diffusion channel. We suggest that two adjacent Al layers in between Cu layers in pre-θ′-1 precipitates have such a fast diffusion channel. P-II in Fig. 2f is still in the pre-θ′-1 phase, given the dark contrast in the plane between the two Al layers and the absence of lattice contraction. When precipitates transform to later stages, the fast diffusion channels are most likely absent, since in those cases we do not observe a fast removal of precipitates when precipitates meet.

The transformation from the small clusters to θ′ has only one large energy barrier: the transformation of pre-θ′-2 to θ′. The earliest stage of the precipitates with an extended regular structure (pre-θ′-1) consists of three parallel Cu planes along Al_(001)_, separated by two Al planes. In the evolution to θ′, the two outer Cu planes remain present during the whole process, indicating they are quite stable. For the pre-θ′-1 phase, the spacing of the Cu planes is constrained by the surrounding matrix, such that the Cu plane - Cu plane distance is exactly 1.5 times the unit cell axis of Al. When the precipitate grows along the plate direction, the lattice of the precipitate can relax and the lattice contracts (to pre-θ′-2) since the Cu atom is smaller than the Al atom. This relaxation can occur without a significant energy barrier. The transformation to the θ′ phase requires substantial rearrangements of the atoms, as can be seen from Fig. 4e–h. In order to allow all atom displacements, the lattice has to expand during this transformation from pre-θ′-2, with its almost FCC lattice, to θ′ with its distinctly different structure. This transformation requires a significant amount of energy, and can only occur if the precipitate is large enough to overcome the energy barrier. It is noteworthy to mention that GP zones and other types of precipitates may form at an ageing temperature below 140 °C. In this work, we have mainly studied the direct formation of θ′-precipitates at atomic scale as a typical example. The strong correlation in morphological features of the precipitates from *in-situ* and bulk heated samples (see part 7 of Supplementary Information and Fig. S13) suggest the growth kinetics from this study can be extended to that of bulk specimens.

In conclusion, we showed that *in-situ* TEM provides a versatile platform to study the complex microstructural evolution in metal alloys: for example, during the multi-step heat treatment frequently used in industry. Because we could return to the starting situation by a solution heat treatment, we managed to do repeated studies on (almost) the same sample (and its local microstructure) at various temperatures. At 140 and 160 °C, we observed that the oval plate-like precipitates can merge very quickly when they meet, which does not occur at higher temperatures. We attribute this to fast diffusion paths in the low temperature early stage precipitates that do not exist long enough at higher temperatures. Some precipitates were found to shrink on one end while growing faster at the other end, thus showing an effective displacement during growth. At all annealing temperatures, most precipitates are surrounded by Cu rich areas. Furthermore, we could follow the evolution of a single precipitate through the various pre-θ′ stages, allowing the construction of a transition model.

## Methods

### Materials and Specimen Preparation

A high purity Al-Cu 5.7 wt.% alloy was used in this study. The amount of impurity elements such as Mg or Si was controlled to be less than 0.02 wt.%. The as-received materials were cold-rolled sheets with a thickness of 1 mm. A sheet was then cut into pieces of 10 × 10 × 1 mm^3^. The TEM specimens of dimensions in the order of 5 μm × 10 μm and thickness of 220–250 nm (see Figure S2c in Supplementary Information) were prepared using the FEI Strata dual beam 235 focused ion beam (FIB/SEM). In order to exclude the effect of grain boundary on the precipitation, the FIB lamella for the *in-situ* study was prepared from a single grain of the alloy sample.

### *In-situ* S/TEM imaging (MEMS based systems)

These TEM lamellae were transferred onto a MEMS micro-heater chip, over which the lamella was suspended on holes in the SiN membrane. The chip was mounted in a DENSsolutions wildfire D6 double-tilt TEM heating holder (see Figure S2 in Supplementary Information for images of MEMS chip). The *in-situ* heating experiments were performed in a FEI Titan microscope equipped with a spherical aberration corrector, operated at 300 kV. This microscope allows HAADF-STEM imaging with a resolution of 0.14 nm, which is sufficient for the resolving the Al-atom columns of the Al-matrix (*fcc* crystal structure, unit cell length: 0.405 nm) in [001] direction. All images were recorded with the electron beam parallel to [001]_Al_. The heat-treatment procedure (T6) adopted in industry to render age hardening was conducted on the TEM specimens while being inside the TEM. Firstly, the specimen was heated to 520 °C to dissolve all the precipitates and constituents. It has to be noted that the solution treatment doesn’t need to last 1 h (2 min in our case), as the homogeneous solid solution was much easier to be achieved in the thin TEM specimen than in bulk materials. Immediately after quenching to 20 °C, the specimen was heated to the set ageing temperature (140–200 °C) within 15 seconds. As the MEMS heater allows a heating rate higher than 100 °C/s, both the temperature increase and decrease can be very fast. The drift during heating at ageing temperature was so small that it did not influence the quality of the STEM images and the temperature fluctuation was less than 0.05 °C. Note that the drift stability of the specimen is of crucial importance for achieving atomic resolution images during *in-situ* heating. For high-resolution STEM imaging, the size of the HRSTEM micrograph was 1024 pixels × 1024 pixels, with one pixel size of 15.0 pm and a dwell time of 50 μs.

## Electronic supplementary material

Supplementary Movie S1

Supplementary Movie S2

Supplementary Movie S3

Supplementary Movie S4

Supplementary Movie S5

Supplementary Movie S6

Supplementary Information
