# Supplementary material for: In-situ STEM imaging of growth and phase change of individual CuAlX precipitates in Al alloy
Source: Sci Rep. 2017 May 19;7:2184. doi: 10.1038/s41598-017-02081-9 (PMC5438361; doi:10.1038/s41598-017-02081-9)
Supplement: Supplementary file 7 — Supplementary Information [file 41598_2017_2081_MOESM7_ESM.pdf]

Supplementary Information for

In-situ STEM imaging of growth and phase change of individual $CuAl_X$ precipitates in Al alloy

Authors: Chunhui Liu[1, 2], Sairam K Malladi[1,5], Qiang Xu[1,3], Jianghua Chen[2], Frans D Tichelaar[1], Xiaodong Zhuge[4] and Henny W Zandbergen[1]*

Affiliations:

[1]Kavli Institute of Nanoscience, Delft University of Technology, 2628 CJ Delft, The Netherlands.

[2]Center for High Resolution Electron Microscopy, College of Materials Science and Engineering, Hunan University, 410082 Changsha, China.

[3]DENSsolutions,Delftechpark 26,2628 XH Delft, The Netherlands.

[4]Computational Imaging (CI), Centrum Wiskunde & Informatica (CWI), Science Park 123, 1098 XG Amsterdam, The Netherlands.

[5]Department of Materials Science and Metallurgical Engineering, Indian Institute of Technology Hyderabad, Kandi, Sangareddy – 502285, Telangana, India.

*Correspondence to: H.W.Zandbergen@tudelft.nl

This PDF file includes:

1. Alloy material and age hardening treatment on bulk samples
2. More details on in-situ STEM studies and Image Processing:
3. Growth kinetics of the precipitates at different temperatures
4. Mechanism controlling precipitate growth
5. Shrinking kinetics and process
6. Density functional theory calculation of precipitate structures in Al-Cu alloys
7. Three-dimensional visualization of the precipitate structure
8. Compositional analysis of the precipitates
9. Electron beam effect
10. Supplementary movie captions

Other Online Supporting Material for this manuscript includes the following: Movies S1 to S6

1. Alloy material and age hardening treatment on bulk samples:

An Al-Cu 5.7 wt. % alloy was used in this study. The as-received materials were cold-rolled sheets with a thickness of 1 mm. A sheet was then cut into pieces of 10×10×1 mm^3. These pieces were solution treated at 520 °C for 1 h in an oil bath and were water-quenched to room temperature and next annealed at 120-200 °C. Vickers micro-hardness tests were performed with a digital hardness tester at a load of 4.9 N with a dwell time of 10 s. Fig. S1 shows the hardness-time curves of the samples aged at 160 °C and 180 °C. The alloy shows a typical strong age hardening. Though the ageing kinetics are accelerated significantly at higher temperatures, similar trend at various temperatures are observed: the hardness increases rapidly at the beginning, it continues to increase gradually after a slight softening until the peak hardness is reached, and then a plateau is formed. The rapid hardness and softening normally correspond to the formation and dissolution of clusters or GP zones[1,2], respectively. The gradual hardness increase represents the nucleation and growth of the effective strengthening phase, θ', in this case[3], while the plateau indicates the microstructure has reached the steady state.

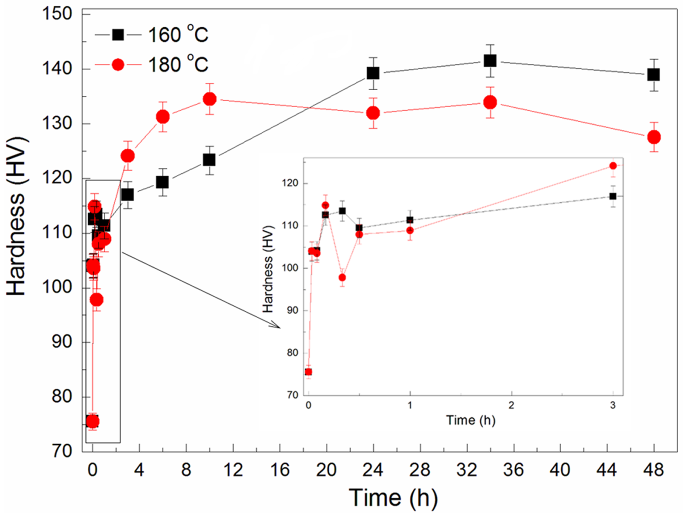

Figure S1: The evolution of hardness as a function of time during artificial ageing. The plot indicates the peak hardness, 135~140 HV, is much higher than the as-quenched state (~75 HV). This considerable strength increment results from the precipitates formed during artificial ageing.

2. More details on in-situ STEM studies and Image Processing:

The in-situ TEM studies were carried out using a DENSsolutions wildfire D6 double-tilt TEM heating holder. The specimen preparation has been carried out using the standard protocols and scripts followed while using an FEI Strata Dual-Beam 235 SEM-FIB. As this instrument is not equipped with an in-situ micromanipulator, the as-prepared thin TEM lamellae were transferred onto the heating chip using a

pneumatic micromanipulator with glass-capillary tips under an optical microscope. The dimensions of the specimen were 15 µm × 6 µm × 200 nm. The specimen thickness was intentionally 200 nm since it should be more than the mean-diffusion length, which is around 160 nm for Cu in Al matrix at 200 °C for a heating time of 24 hours. Fig. S2 shows the picture of Pt heating spiral, embedded in SiN membrane and the lamellar TEM specimens suspended over holes in the SiN membrane.

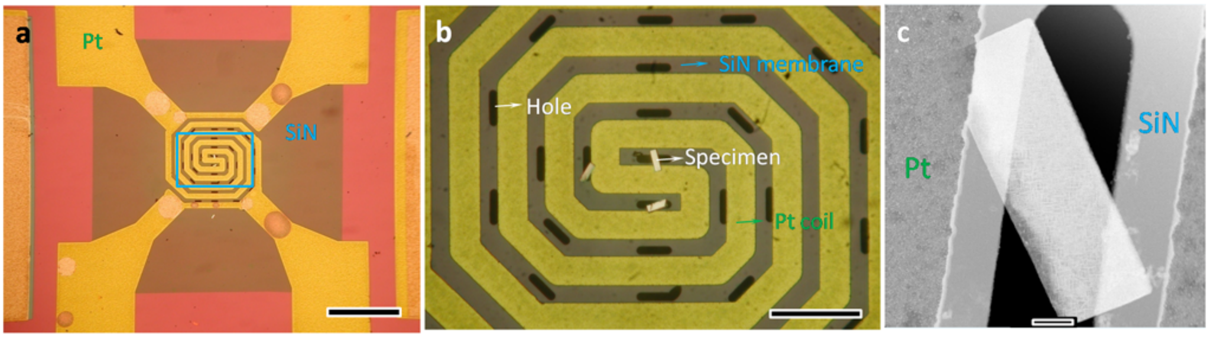

Figure S2: FIB lamella on thin membranes of MEMS based heating chip. (a-b) Optical micrographs, the right picture is magnified central part of the left one; (c) Low magnification ADF-STEM image of FIB lamella. Scale bars (a), 200 µm, (b), 50 µm, (c), 2 µm.

2.1 High-Angle Annular Dark-Field Scanning Transmission Electron Microscopy (HAADF-STEM) imaging

In HAADF-STEM imaging mode, the intensity scales approximately with Z[24,5]. As Cu (Z=27) has a higher atomic number than Al (Z=13), the precipitates containing a lot of Cu solutes appear with higher intensity in HAADF-STEM images in comparison with the Al matrix. The raw atomic-resolution images shown in the paper (Fig. S3a) were processed by applying a mask in the Fourier transform of the original images. This method reduces the noise without introducing artificial information as shown in Fig. S3b.

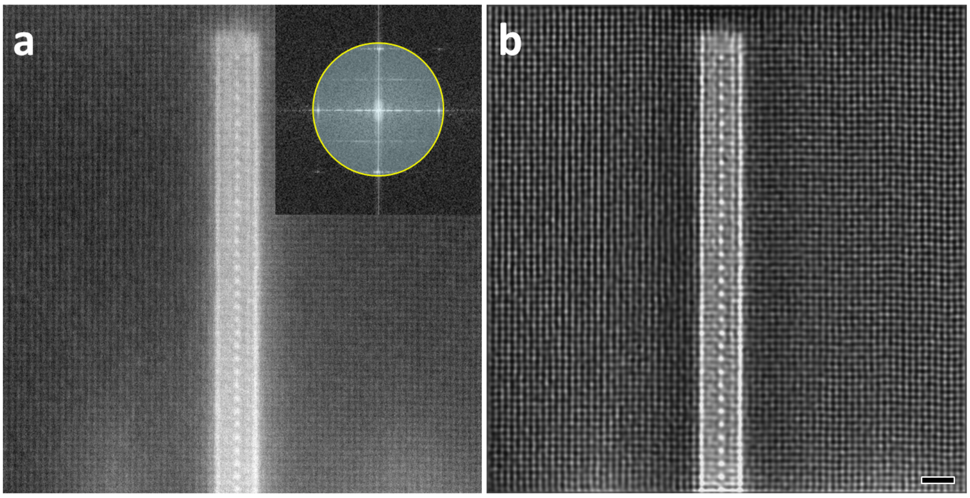

Figure S3: (a) the original image of Fig. 4c, the inset is the Fast Fourier Transform (FFT) superimposed with a circular mask;(b) the filtered image used in Fig. 4c; actually, this image is acquired by conducting inverse FFT on the masked FFT pattern in (a). Scale bar, 1 nm.

2.2 Data analysis and statistics using Image J

The process used to acquire the length of one of the precipitates (marked P1 in Fig. 1e) from *in-situ* STEM movie based on the method used in reference 21 of the main text is described in this section. Fig. S4a shows an ADF-STEM frame taken from the end of the movie S2. In order to enhance the contrast of the edges of the precipitate, the image stack is first duplicated and processed using a Gaussian blur (sigma radius 4.00). Then the original image stack is divided by the processed image stack to produce a stack of images with sharp precipitate edges. Subsequently, an orthogonal image (through the image series) with the time series on the X-axis and the precipitate length on the Y-axis from each of the precipitates is procured.

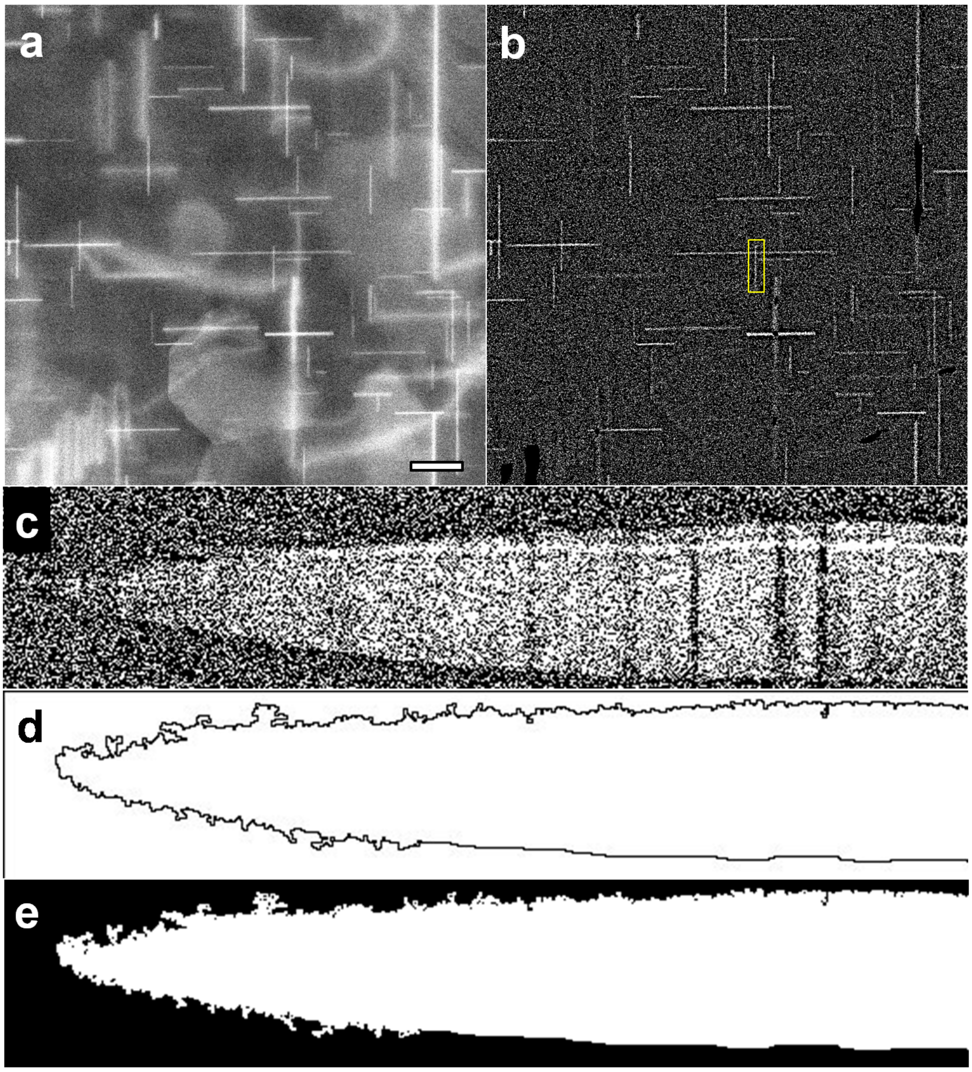

Figure S4: (a) Final frame of an ADF –STEM movie (movie S2). Scale bar, 50 nm.(b) Final frame of the same movie after image processing. (c) Orthogonal view, YZ(length-time) revealing the growth trajectory of the precipitate by a rectangle in (b). (d) Outline map of (c) generated by setting appropriate thresholds. (e) Bitmap image where all the pixels outside the outline are set to 0 and inside to 1. From (e), the summation of pixels along the y-axis now gives the change in length of precipitate with time.

Fig. S4c shows the growth trajectory of a precipitate indicated by the yellow rectangle in Fig. S4b.Note that this image actually reveals also the growth/shrinkage (any variation in the length) of the precipitate in time. All the plots shown in Fig. 1h were acquired in this way. To determine the length of the precipitate, first a bare outline map, Fig. S4d, is extracted from the 'Analyse Particles' plug-in of ImageJ using an appropriate threshold level. Then, all pixels outside the particle growth trajectory are set to 0 and the pixels inside are set to 1 to generate a bitmap as shown in Fig. S4e. The length of the precipitates is then calculated by adding the pixels along the vertical axis and multiplying with the pixel size of the ADF-STEM images.

3. Growth kinetics of the precipitates at different temperatures

From movies S1-S3, the changes in lengths of a few precipitates with ageing time were analysed, as shown in Fig. S5. The data of precipitates formed during ageing at 200 °Care not given here because their relatively large sizes could imply intersection with the surface. From the 3-D reconstruction (see below) based on STEM-tomography, the thickness of the TEM sample was determined to be about 220 nm, which was smaller than most of the precipitates formed in the sample aged at 200 °C.

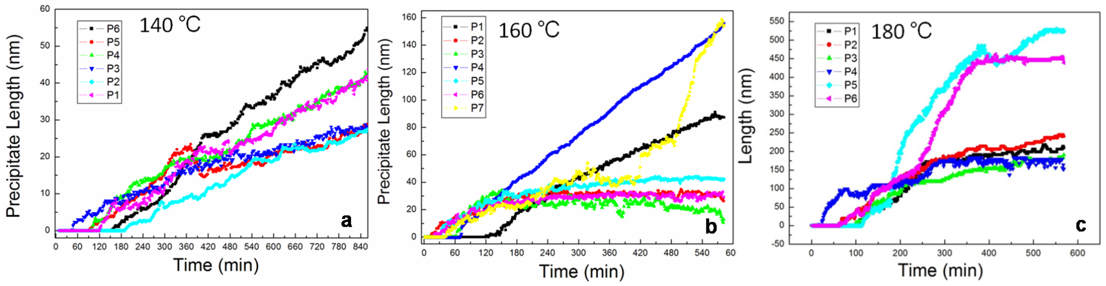

Figure S5: The change of precipitate length as a function of time for the TEM samples heated *in-situ* at(**a**) 140 °C; (**b**) 160 °C; (**c**) 180 °C. The data are extracted from Movies S1-S3, respectively.

4. Mechanism controlling precipitate growth

There exist several theories on the precipitate coarsening and most notable are the ones based on diffusion of the alloying elements[6]. Recently, models based on a combination of mesoscale phase-field method with atomistic approaches have been developed too, predicting the morphology of θ' precipitates in Al-Cu alloys[7,8]. In this study, we do not validate the model for morphology based on phase-field approach, however, we observe that the precipitate growth is diffusion-controlled.

The growth trajectories for twenty random precipitates in the field of view were extracted from Movies S1-S3, as shown in Fig. 1 and Fig. S5. For each of the precipitates, by synchronizing time of nucleation as t = 0, the length of a precipitate has been plotted as a function of $t^{½}$. Seventeen precipitates show a linear relationship between precipitate length and $t^{½}$ as shown in Fig. S6a and Fig. S6b.These follow the volume-diffusion controlled growth of precipitates in metallic alloys[9,10] described by the relation:

$$½L(t) \propto 2(\beta\ D)^{½}\ t^{½} \text{ or simply } L(t) \propto A\ t^{½} \quad (1)$$

where the β is, a parameter related with the super-saturation of solutes, D is diffusion coefficient.

The slope values (A) for length change curves of different precipitates at various temperatures were

determined by fitting the data from the previous section. The value ranges are shown in Table S1. Obviously, the precipitates grow faster with increasing ageing temperature.

A small fraction of precipitates (3 out of 20) show a length change linear with t (Fig. S6c) following a relationship as:

$$L(t) \propto t \qquad (2)$$

From the morphology of these precipitates and from the location determined from tomography studies, we conclude that these precipitates grow faster as they are located near the surface, also as observed by Ferrante & Doherty[9]. This kind of growth was observed to occur at higher ageing temperatures (typically beyond 180°C).The exception is when a precipitate interacts with a dislocation as shown by precipitate P3 in Fig. 1, the precipitate growth is assisted by volume-diffusion, till the precipitate interacts with the dislocation after which its growth rate is accelerated as shown in Fig. 1h

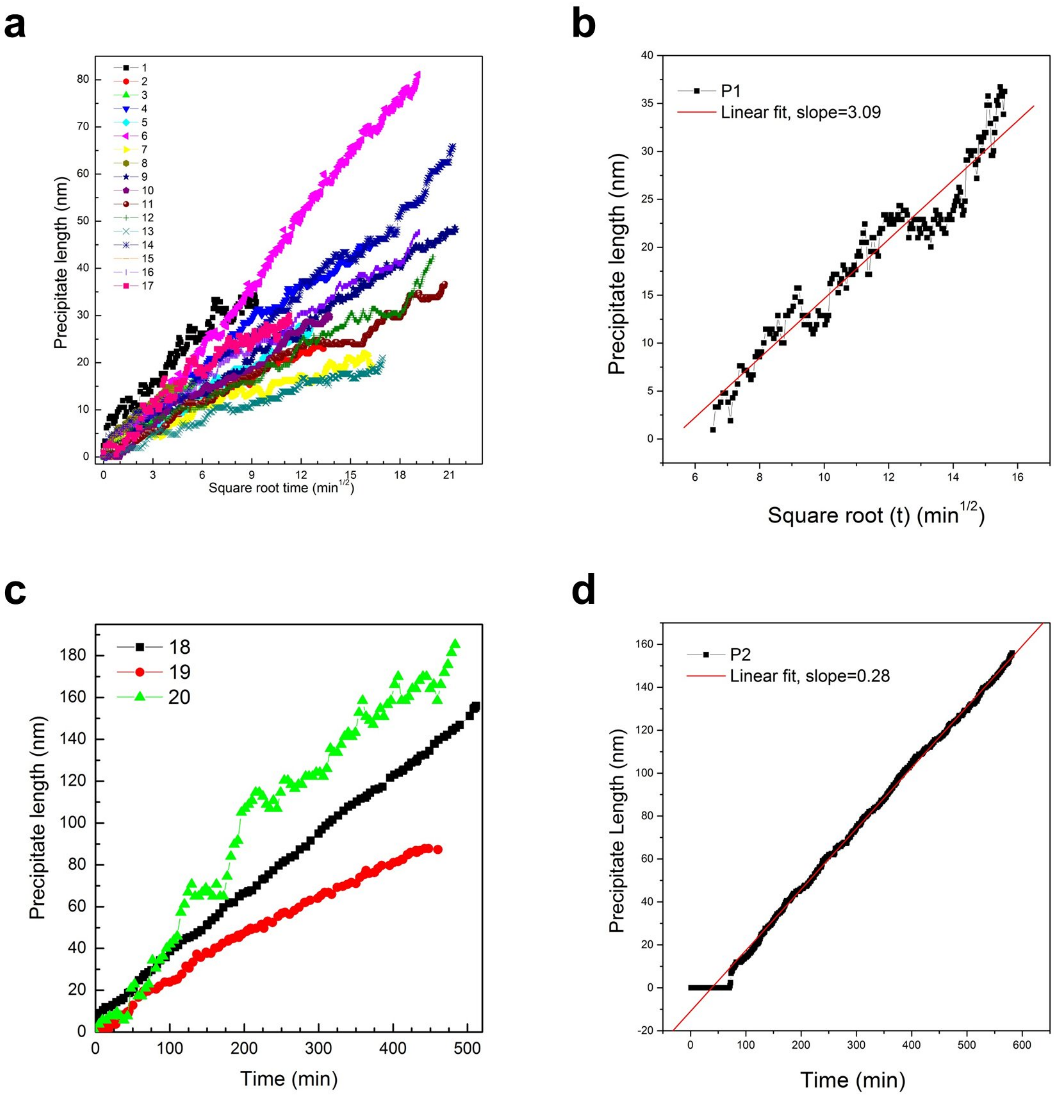

Figure S6: Liner fits for precipitate length versus time: (**a**) seventeen precipitates show a linear relationship between length and $t^{1/2}$ and note that (**b**) the length of precipitate P1in Fig. 1 varies linearly as a function of $t^{1/2}$; whereas in (**c**) three precipitates showing the variation of length as a linear function of t and (**d**) precipitateP2in Fig. 1 varies as a function of t.

Table S1: Slope values determined through analysis of the data at various temperatures

Temperature (℃)	A (nm·min$^{-1/2}$)
140	1.19 ~2.16
160	2.74 ~3.23
180	5.42 ~11.87

5. Shrinking kinetics and process

A remarkable observation in this study is the relatively fast shrinkage of one of the precipitates when it comes in contact with a larger growing precipitate in the vicinity, as shown in Fig. S7 a-e. Fig. S7 a-e shows the as-acquired HR-STEM images revealing the shrinkage of plate-shaped precipitates II in Fig. 3. It is evident that the thickness remains unchanged during the entire process of shrinking while the length reduces significantly. The area of intersection is brighter than either of the two precipitates, indicating an overlap in the projection direction. A proposed position of precipitates I and II is illustrated in Fig. S7f

The initial structure of the precipitate is composed of alternating Cu and Al planes, with the intensity of the former brighter than the latter in HR-STEM image, as proved by the grey level intensity analysis of the lattice planes in the precipitate shown in Fig. S8. The data points for Fig. S9 are actually the average values corresponding to the five bright peaks and the neighbouring peaks in Fig. S8.The variation in intensity ratios between the Al planes and Cu planes in the precipitate can be observed and indicates the initial structure is altered only at the last shrinking stage.

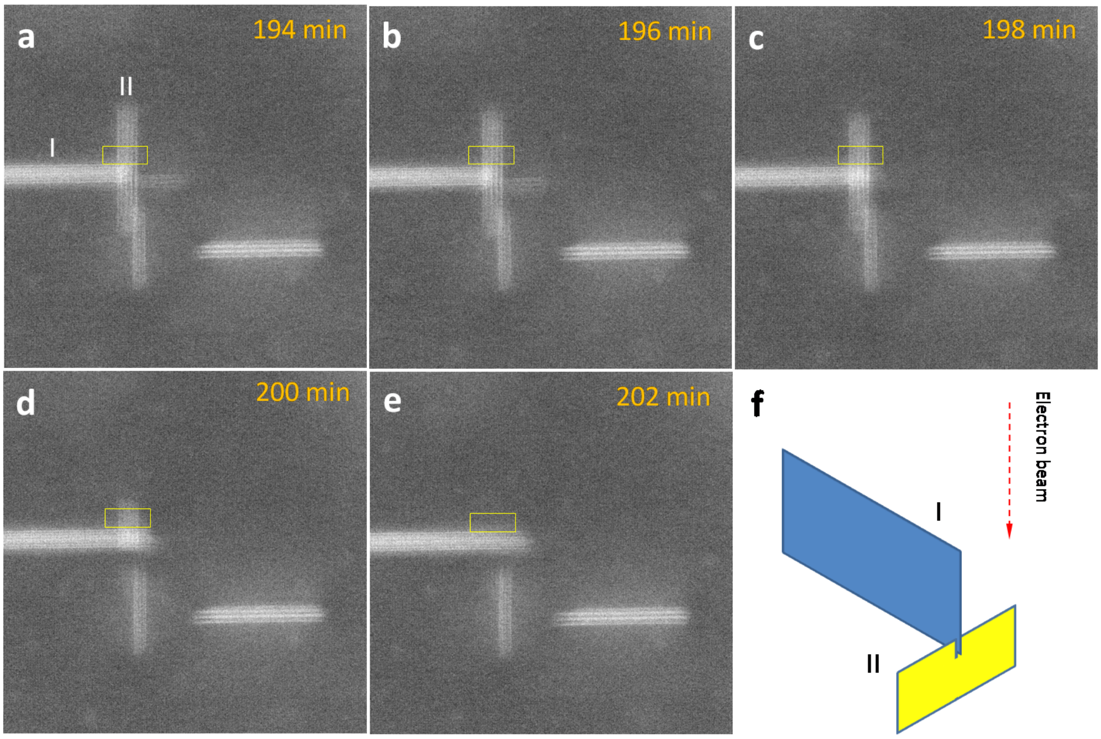

Figure S7: (**a-e**) Sequential ADF-STEM images (extracted from movie S5) showing the merging process of precipitates in Fig. 3; (**f**) The three-dimensional geometrical positions of intersected precipitates I and II.

The decrease in the length of the precipitate with time was estimated by calculating the relative change in the intensity from the HAADF-STEM image which is proportional to the mass and thickness of the sample. For a single phase, the intensity of HAADF-STEM image (I) is proportional to the thickness (T) of the sample. Note that the thickness of the specimen has not changed throughout the study. Consider that 'M' is the contribution per thickness from Al matrix to the intensity and 'P' is the contribution from the precipitate (Cu layers) per thickness. In case of just the matrix contributing to the intensity of STEM image, the total intensity (I) of the image can be written as:

$$T \cdot M = I \qquad (3)$$

Whereas for the regions including a precipitate, the contribution factors from both the precipitate (P) and the matrix (M) are prominent. The intensity of a region including a precipitate (I_P) can then be written as:

$$(T-T_p) \cdot M + T_p \cdot P = I_p \qquad (4)$$

where T_p is the length of the edge-on precipitate along the thickness direction.

Combining equation (3) and (4):

$$T_p \cdot (P-M) = I_p - I \qquad (5)$$

Assuming P, M and I don't change during this short process, we can easily estimate the change of T_p with respect to the initial length by measuring I_p of each image in Fig. S7. The determined dimensional change along the thickness direction is shown in Fig. S9. The results reveal the asynchronous nature of the shrinking in different dimensions of the precipitate. Only after the size parallel to the normal axis of precipitate-I is reduced to a very small value, the precipitate starts shrinking in another orthogonal axis.

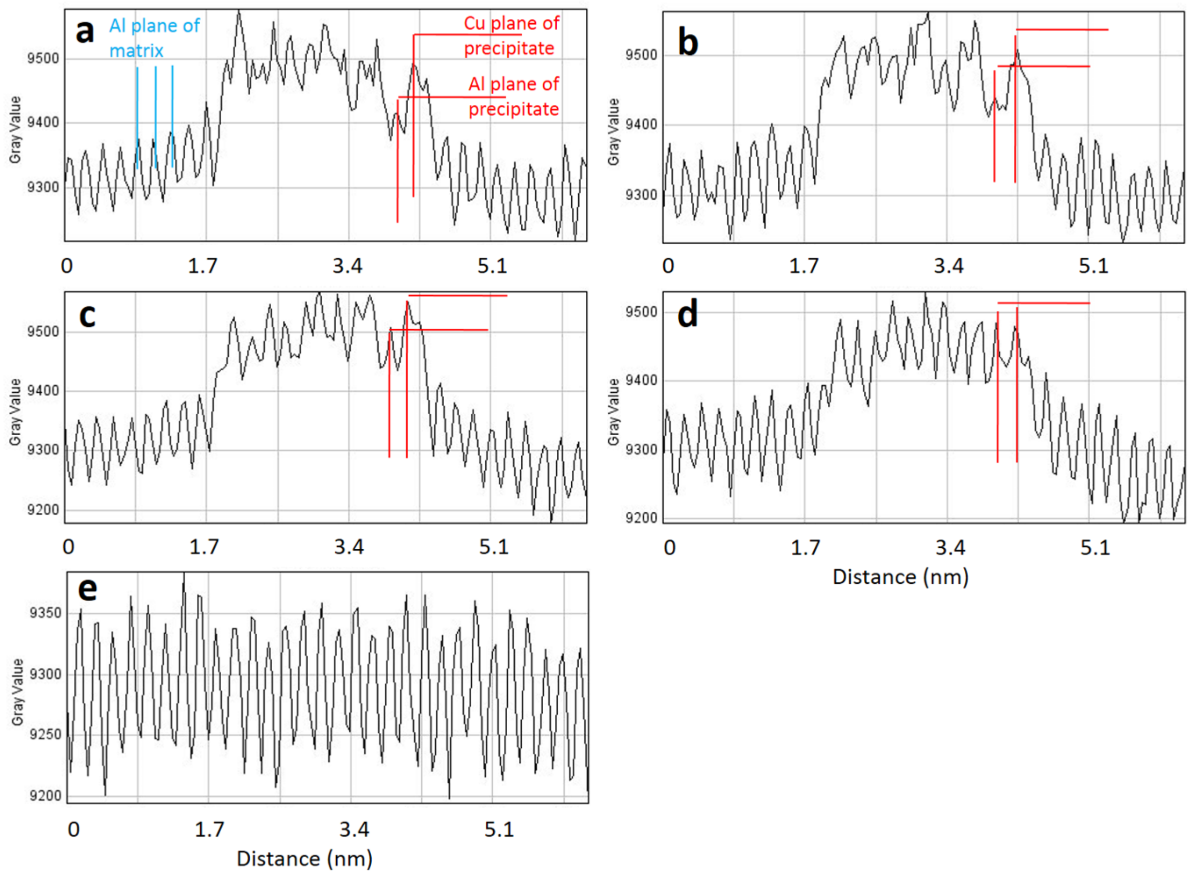

Figure S8: Grey value intensity profile plots along the box in Fig. S7: the variation in intensity of the atom planes in the precipitate is highlighted to show the change in Cu plane to Al plane ratio. ImageJ software was used for the analysis.

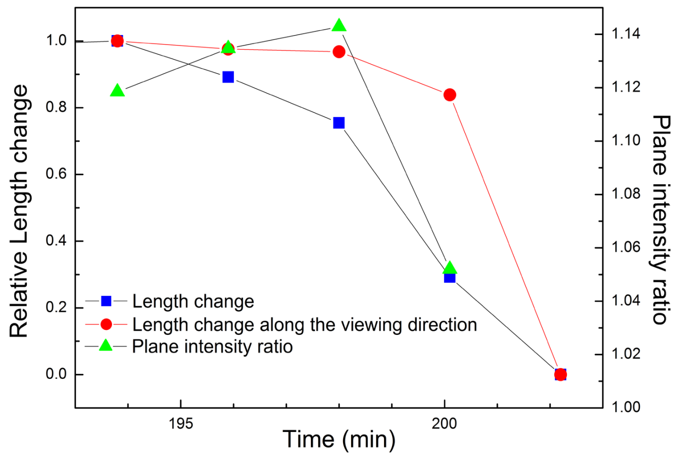

Figure S9: The dimension change of the shrinking precipitate II in Fig. 3. The length change was obtained firstly by measuring the lengths of the shrinking precipitate in Fig. S7, and then dividing them by the initial length. The plane intensity ratio represents the grey value intensity of the Cu planes (bright lines) to that of the Al planes (dark lines). The structures of the precipitates formed in the investigated alloy are composed of alternative Cu and Al planes, whose intensity ratio is larger than one. Therefore, the high ratio value thus indicates the precipitate keeps its original structure before disappearance.

6. Density functional theory calculation of precipitate structures in Al-Cu alloys

First-principles calculations were performed to investigate phase stability of precipitate structures formed at various stages. Supercells shown in Fig. S10 were used to model the precipitate embedded in the Al matrix. The calculations were performed using the plane-wave based Vienna ab initio simulation package (VASP)[11-13]. The interaction between ion and core electrons was described by the projector augmented wave (PAW) method, and plane waves with an energy cut-off of 250 eV are used to expand the Kohn-Sham(KS) wave functions. The generalized gradient approximation (GGA) for the exchange and correlation functional is employed with PW91 scheme. We used 15×15×10 k-points mesh generated by the Monkhorst-Pack scheme. With these input parameters (e.g., energy cut-off, *k*-point sampling and supercell size), the formation energies converge to better than 10 meV.

The formation enthalpy, ΔH, is defined as the difference between energies of a Cu-enriched precipitate structure and the isolated impurities. In the present work, we define the formation enthalpy with respect to the bulk Al and Cu atom dissolved in Al matrix as

$$\Delta H_{SS}^{form}(Al_xCu_y) = H(Al_xCu_y) - xH(Al^{fcc}) - yH(Cu^{sub}) \quad (6)$$

where x and y are the numbers of atoms in the supercell, and $H(Al_xCu_y)$, $H(Al^{fcc})$,and $H(Cu^{sub})$ are the enthalpies of Cu-enriched precipitate, *fcc* Al, and substitutional Cu in the Al matrix, respectively. The enthalpy of a substitutional Cu in a relaxed Al supercell is given by

$$H(Cu^{sub}) = H(Al_{107}Cu) - \frac{107}{108}H(Al_{108}) \quad (7)$$

The reliability of this method was established by confirming from some of the existing precipitate structures. Low enthalpy and low mismatch with matrix indicate favourable structure. All the optimized crystal lattice parameters except *a* of transient stage (shift) are smaller than corresponding Al lattice parameters, as shown in Table S2. This indicates a lattice contraction when Cu is enriched at a specific site to form precipitate.

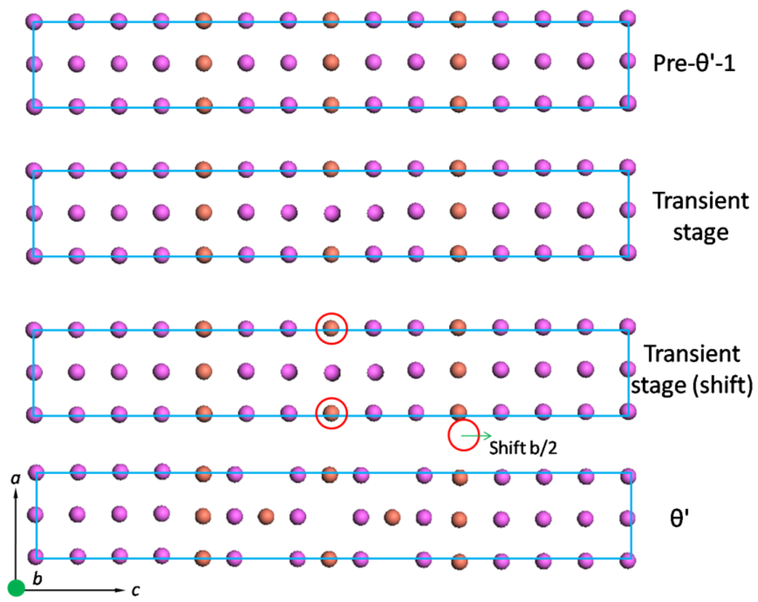

Figure S10: The supercells built for DFT calculations: Both the Al matrix and the precipitate are included in the calculation. The atom positions overlaid with a cycle are shifted by b/2 relative to the transient stage.

Table S2 Optimized crystal lattice parameters of various precipitates through DFT calculations: + and - represent expansion and contraction, respectively, relative to the Al lattice.

parameters	*a*	*b*	*c*	Formation enthalpy
Al lattice	0.405 nm	0.405 nm	2.835 nm	0
Pre-θ'-1	0.398 nm (-)	0.398 nm (-)	2.690 nm (-)	-0.01433 ev/atom
Pre-θ'-2	0.398 nm (-)	0.398 nm (-)	2.690 nm (-)	-0.01433 ev/atom
Transient phase	0.398 nm (-)	0.398 nm (-)	2.725 nm (-)	-0.00867 ev/atom
Transient phase (shift)	0.420 nm (+)	0.395 nm (-)	2.710 nm (-)	0.0712 ev/atom
θ' (with extra Cu)	0. 404 nm (-)	0.404 nm (-)	2.730 nm (-)	-0.04779 ev/atom

The conclusions regarding the lattice expansion/contraction in the main paper are based on the analysis from Fig. S11. Plots of grey value intensity profiles along the precipitate in Fig. 4 (all four stages during growth) were extracted as shown in Fig. S11. The Al lattice was taken as a reference to scale the distance between the edge Cu planes. The large positive value of formation enthalpy of transient stage (shift) in Table S2 suggests this structure is unfavourable. Note that a model on the transient stage (shift) cannot be obtained from the STEM image, because of the unclear structure, which also reflects a variation in atom positions along the viewing direction. The gradual decrease of formation enthalpies from -0.01433 ev/atom to -0.04779 ev/atom for pre-θ'-1, transient stage and θ' reveals the transformation from pre-θ'-1 is a reasonable kinetic pathway.

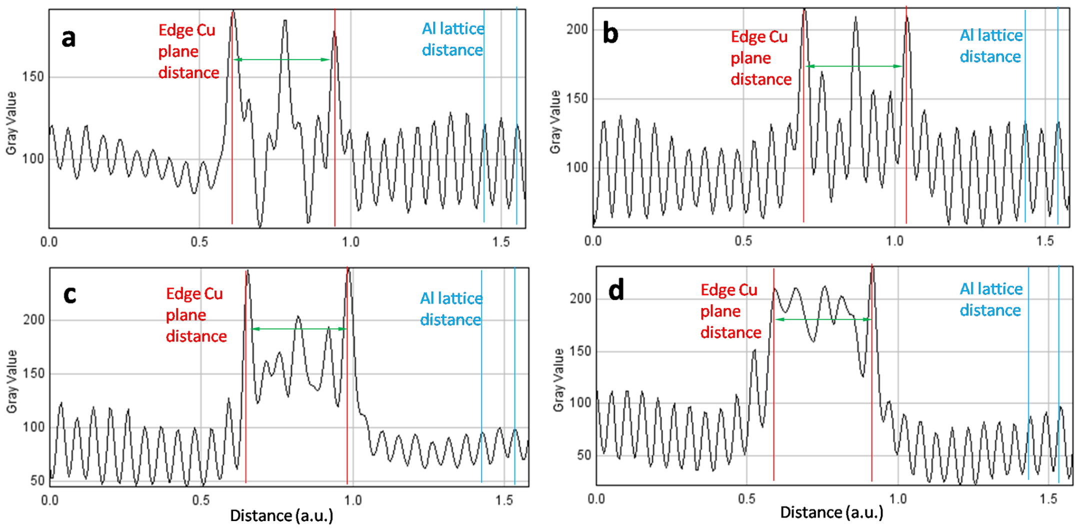

Figure S11: Grey value intensity profile plots along the precipitate in Fig. 4a-d: The Al lattice far from the precipitate was used as the reference in each image to measure the edge Cu plane distance. ImageJ software was used for the analysis.

7. Three-dimensional reconstruction of the precipitate structure

STEM tomography was performed to obtain three-dimensional (3-D) information of the precipitates formed in the FIB lamella. The specimen was tilted over a range of ±45°and ADF-STEM images (2-D projections) were acquired in steps of 1°. In this study, the discrete algebraic reconstruction technique (DART)[14]was employed to reconstruct the 3-D map of the precipitates from the tilt-series. The main purpose of this reconstruction was not only obtaining a 3-D distribution of the precipitates, but also to eliminate the influence of the surface such that the growth kinetics of the plate-like precipitates can be extended to that of a bulk specimen.

Fig. S12 and movie S6 reveal that plate-like nano-precipitates are distributed uniformly and are formed inside the TEM specimen. Although a few large intermetallic particles are observed at the specimen surface, the measured lengths of the plate-like nano-precipitates and the specimen thickness suggest that these Al-Cu rich (θ') precipitates originate inside the sample. Shown in Fig. S13are the ADF-STEM images of the precipitates formed in both in-situ heated sample and bulk sample. All three variants lying on equivalent $\{100\}_{Al}$ planes could be observed. The morphology, precipitate-matrix orientation relationship and sizes of the precipitates in in-situ heated sample are comparable to those in bulk sample. These observations from the tomography and the strong correlation in length of the precipitates from *in-situ* and *ex-situ* heat-treatment studies after 8 h of ageing suggest the growth kinetics from this study can be extended to that of bulk specimens.

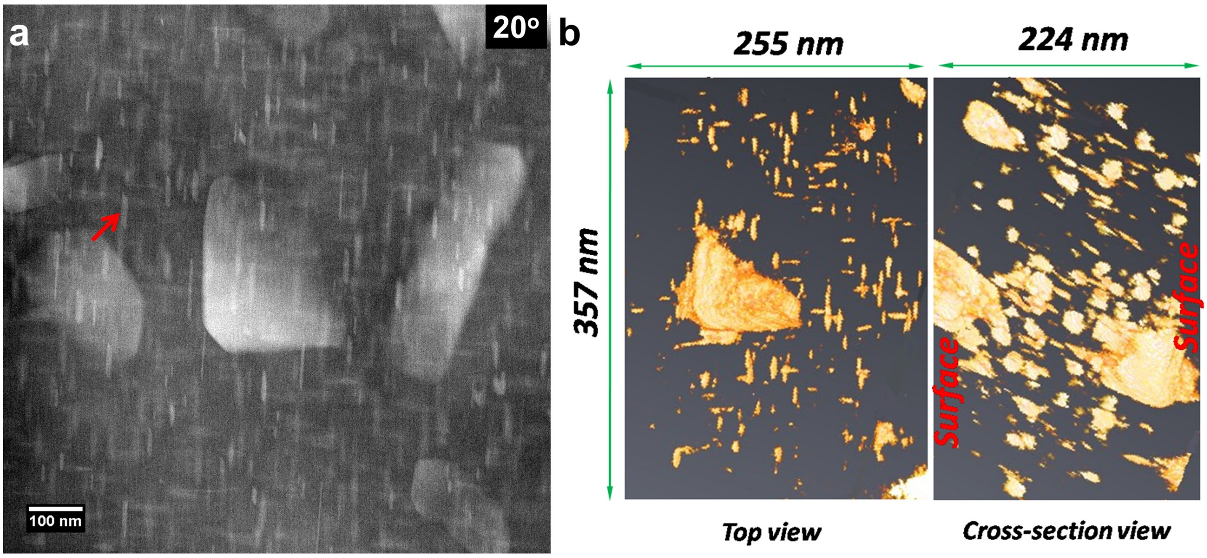

Figure S12: (**a**)STEM-ADF image obtained at a tilt of 20° revealing the elongated plate type θ' precipitate indicated by the red-arrow. These precipitates were grown on heating the TEM sample 5 h at 160 °C. From a series of 91 such STEM-ADF images, the 3-dimensional reconstruction has been carried. (**b**) Images extracted from movie S6, showing the 3-dimensional reconstruction of the plate-like nanoprecipitates. These precipitates are distributed uniformly inside the TEM sample through the thickness. The few large particles are intermetallic particles formed at the sample surfaces.

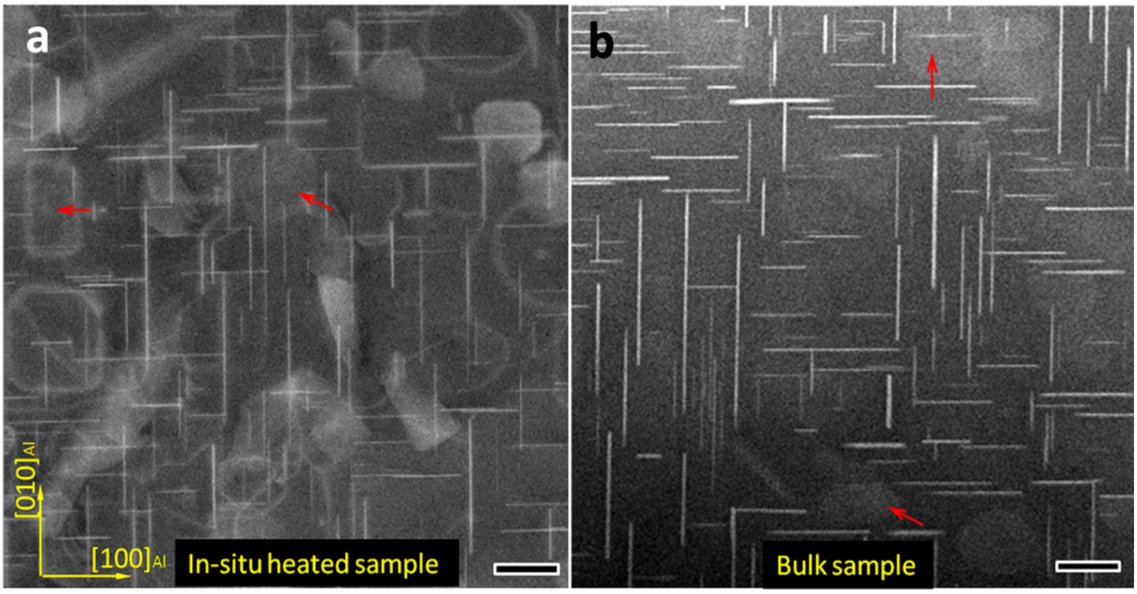

Figure S13: ADF-STEM images showing the morphologies of the precipitates in(**a**) sample in-situ heated for 8 h at 200 °C; (**b**) bulk sample artificially aged for 8 h at 200 °C. Arrows point to face-on precipitates embedded in the matrix. Perpendicular bright needles represent cross-sections of edge-on plate-shaped Cu-rich precipitates. Images recorded with the electron beam along $[001]_{Al}$ axis. Scale bars (**a-b**), 200 nm

.

8. Compositional analysis of the precipitates:

Although a high-purity alloy was used for this study, we carried out EDX mapping to analyse the composition of the precipitates. The STEM images and the corresponding EDX maps were acquired on an FEI TITAN operated at 300 kV equipped with an Oxford Instruments X-Max detector. The specimen was heat-treated in-situ at 160 °C for 8 h to grow the plate-type precipitates. Owing to the thick specimens (200 nm) used in this study and the limitation of the EDX detector, it was only possible to obtain low-resolution EDX maps and the STEM images and corresponding maps are shown in Fig. S14. As can be seen from the Fig. S14, all of the plate-like precipitates are enriched in Cu, indicating θ′ type precipitates.

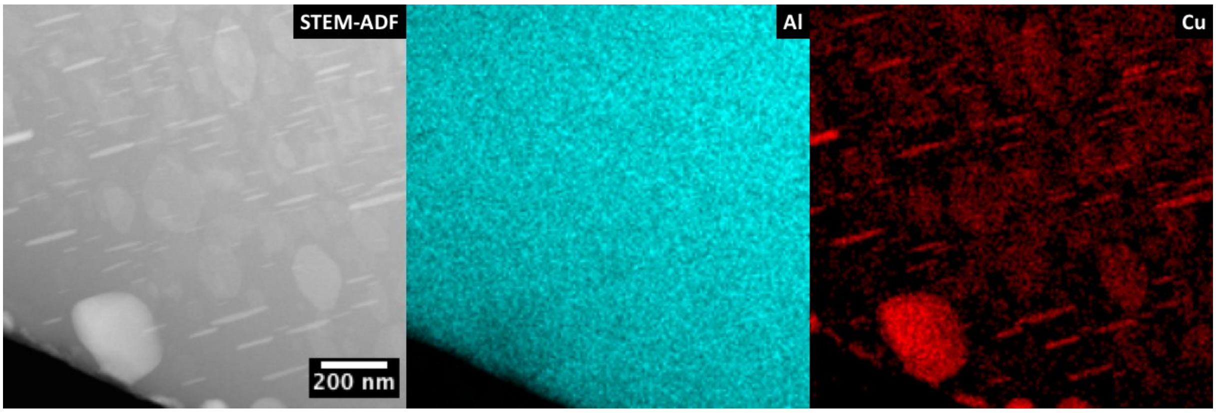

Figure S14: STEM-ADF image and corresponding EDX maps for Al and Cu acquired from a specimen heat treated in-situ for 8 h at 160 ℃. Note that all the plate-type precipitates are enriched in Cu, indicating θ′ type precipitates.

9. Electron beam effect

9.1 Electron-beam heating

Energy transfer from the electron beam to the specimen due to inelastic scattering results for an important part into heat generation. This heat production is balanced in a steady state by heat conductivity to the specimen surrounded by the irradiated area and heat radiation to the environment from both specimen surfaces. The latter term can be neglected according to Egerton in reference 18. The upper limit of the temperature increase can be estimated from the energy input by the electron beam and the heat loss due to conductivity. In reference 18, the temperature increase in a carbon foil exposed to a 5 nA electron beam at 200 kV of ~0.15 nm diameter is estimated to be ~ 1.5 K, independent on specimen thickness. In our case the electron beam of similar diameter in STEM mode at 300 kV in aluminium

with a heat conductivity more than 100 times that in carbon is expected to generate a much lower temperature increase. In addition, the electron beam is not stationary, resulting in even less heat production. The temperature increase under irradiation was also calculated from the similar Fisher's model [15] to be less than 1K.

9.2 Knock-on damage and its recovery

Compared to the high melting point of Al, such a small temperature increase is insufficient to explain the fast shrinkage observed in our experiments. Dynamic displacements of constituent atoms occur under electron irradiation at high voltages, which promotes atom diffusion in crystalline and amorphous phases. The knock-on phenomenon may result from the competition of damage and recovery processes. The accumulation of damage can only occur when the damage rate is higher than the recovery rate. The amount of energy transferred by an electron to the nucleus of an atom can be estimated according to the following equations [18]:

$$E = E_{\max} \sin^2(\theta/2) \tag{10}$$

$$E_{\max} = E_0(1.02 + E_0/10^6)/(465.7A) \tag{11}$$

where θ is the deflected angle of the electron in the field of the atom nucleus, $E_{\max}$ is the maximum energy transferred, E_0 is the incident electron energy (in eV), and A is the atomic mass number. According to the equations, displacement energy and the corresponding threshold value of the incident energy for Al are 17 eV and 180 Kev, while 20 eV and 420 keV for Cu [19].

Therefore, knock-on damage of Al could occur in our case. However, at high temperatures the atoms in the Al sample have a high recovery rate. Thus, the knock-on damage could be significantly reduced when the ADF-STEM images are obtained on the TEM specimen heated at the ageing temperatures.

9.3 Experimental results regarding the effect of the electron-irradiation on the precipitation

To reduce the effect of electron beam, we either lower the electron current density (dose rate) or shorten the dwell time until there is no appreciable difference between the precipitates formed under the electron beam and those formed without electron beam effect (the only stimulus is heating), as shown in Fig. S15. We made a comparison of the precipitates formed in the irradiation area with those formed

in other places. No appreciable difference was found for the samples aged in the temperature range we studied. For recording the time series of the individual precipitate in Fig. 4 at atomic-resolution, the electron beam was therefore blocked during the intervals (several hours) between recording each image to eliminate the electron radiation effect. For the low-magnification movies showing the morphological change, the electron beam effect was not observed in our in-situ experiment. And the difference of precipitation behaviours revealed in our results should be caused by the temperature difference, as the imaging conditions were the same.

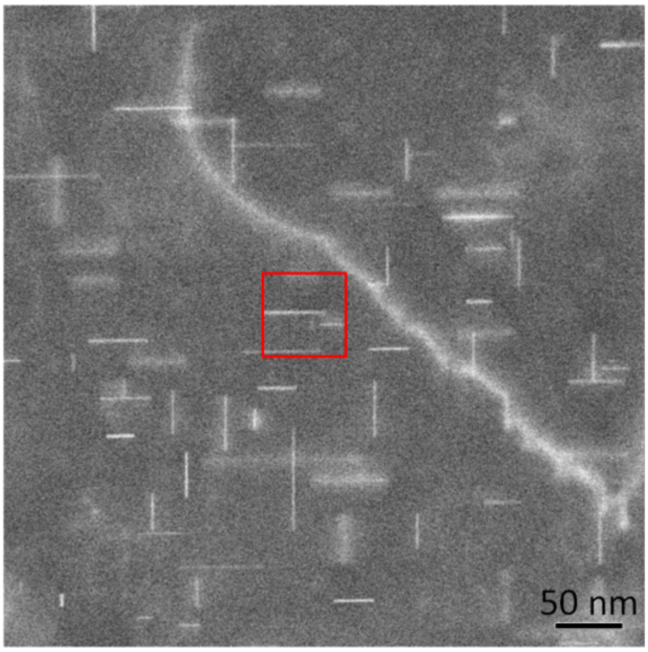

Figure S15: Comparison of the precipitation occurring under electron beam (the region indicated by red box) with that free of electron beam effect (the region outside the red box area). The sample was heated for 5 h at 160 ℃ with the area (Movie S5), marked by the red box, under continuous scanning by electron beam.

10. Supplementary movie captions:

- **Supplementary Movie S1:** Movie showing the nucleation and growth of precipitates in a FIB specimen of Al-Cu alloy heated at 140°C. Each frame is an ADF-STEM image recorded with the electron beam parallel to $<001>_{Al}$ orientation. The time (in hours/minutes/seconds) for which the specimen is held at 140 °C immediately after quenching, is shown on the upper-left corner.
- **Supplementary Movie S2:** Movie showing the nucleation and growth of precipitates in a FIB specimen of Al-Cu alloy heated at 160°C. Images extracted from this movie are also shown in Fig. 1 in the main text, to reveal the evolution of precipitates at this temperature. Each frame is an ADF-STEM image

recorded with the electron beam parallel to $<001>_{Al}$ orientation. The time (in hours/minutes/seconds) for which the specimen is held at 160 °C immediately after quenching, is shown on the upper-left corner.

- **Supplementary Movie S3:** Movie showing the nucleation and growth of precipitates in a FIB specimen of Al-Cu alloy heated at 180°C. Each frame is an ADF-STEM image recorded with the electron beam parallel to $<001>_{Al}$ orientation. The time (in hours/minutes/seconds) for which the specimen is held at 180 °C immediately after quenching, is shown on the upper-left corner.
- **Supplementary Movie S4:** Movie showing the nucleation and growth of precipitates in a FIB specimen of Al-Cu alloy heated at 200°C. Each frame is an ADF-STEM image recorded with the electron beam parallel to $<001>_{Al}$ orientation. The time (in hours/minutes/seconds) for which the specimen is held at 200 °C immediately after quenching, is shown on the upper-left corner.
- **Supplementary Movie S5:** Atomic-scale ADF-STEM movie of the nucleation, growth and dissolution of precipitates in a FIB specimen of Al-Cu alloy heated at 160°C.The time (in hours/minutes/seconds) for which the specimen is held at 160 °C immediately after quenching, is shown on the upper-left corner. The movie is acquired at a rate of 126 seconds per frame. In the first 20 min, some brighter areas appear due to aggregation of Cu solutes. Two of the five aggregates develop into precipitates. Several stages of the growth of these two precipitates are observed, as shown in Fig. 2 in the main text. The change in positions of the two rims of precipitate II is quite remarkable. After 158 min of ageing, a larger precipitate is in contact with a relatively smaller one. Then the latter one is dissolved in several minutes, as detailed in Fig. 3.
- **Supplementary Movie S6:** Three-dimensional rendering of the precipitates in FIB Al-Cu sample in-situ heated for 5 h at 160°C. This tomogram is reconstructed from ADF-STEM tilt-series and is rotated by 360°about the vertical tilt axis at the centre of the reconstructed volume. The two large particles are located respectively at the upper and lower surfaces of the specimen, while the fine plate-like nano-scale precipitates are distributed uniformly in the specimen.

References and notes:

1 S.C. Wang, M. J. S. Precipitates and intermetallic phases in precipitation hardening Al-Cu-Mg-(Li) based alloys. *Int. Mater. Rev.* **50**, 193-215 (2005).

2 L. Liu, J. H. C., S.B. Wang, C.H. Liu, S.S. Yang, C.L. Wu. The effect of Si on precipitation in Al-Cu-Mg alloy with a high Cu/Mg ratio. *Mater. Sci. Eng. A* **606**, 187-195 (2014).

3 L. Bourgeois, N. V. M., A.E. Smith, M. Weyland, J.F. Nie, C. Dwyer. Efficient atomic-scale kinetics through a complex heterophase interface. *Phys. Rev. Lett.* **111**, 046102 046101-046105 (2013).

4 Howie, A. Image contrast and localized signal selection techniques. *J Microsc.* **117**, 11-23 (1979).

5 Pennycook, S. J. Z Contrast STEM for materials science. *Ultramicroscopy* **30**, 58-69 (1989).

6 Ham, F. S. Theory of Diffusion-Limited Precipitation. *J. Phys. Chem. Solids* **6**, 335-351 (1958).

7 Vaithyanathan, V., Wolverton, C. & Chen, L. Q. Multiscale modeling of θ′ precipitation in Al－Cu binary alloys. *Acta Materialia* **52**, 2973-2987, doi:10.1016/j.actamat.2004.03.001 (2004).

8 De Geuser, F., Gable, B. M. & Muddle, B. C. CALPHAD based kinetic Monte Carlo simulation of clustering in binary Al-Cu alloy. *Philosophical Magazine* **91**, 315-336, doi:10.1080/14786435.2010.519354 (2011).

9 M. Ferrante, R. D. D. Influence of interfacial properties on the kinetics of precipitation and precipitate coarsening in aluminum-silver alloys. *Acta Metall.* **27**, 1603-1614 (1979).

10 Starink, M. J. A new model for diffusion-controlled precipitation reactions using the extended volume concept. *Thermochim. Acta* **596**, 109–119 (2014).

11 G. Kresse, J. H. Norm-conserving and ultrasoft pseudopotentials for first-row and transition elements. *J. Phys.: Condens. Matter* **6**, 8245-8257 (1994).

12 G. Kresse, J. F. Efficient Iterative Schemes for ab Initio Total-energy Calculation Using a Plane-wave Basis Set. *Phys. Rev. B* **54**, 11169-11186 (1996).

13 G. Kresse, J. F. Efficiency of ab-initio total energy calculations for metals and semiconductors using a plane-wave basis set. *Comput. Mat. Sci.* **6**, 15-50 (1996).

14 K.J. Batenburg, S. B., J. Sijbers, C. Kübel, P.A. Midgley, J.C. Hernandez, U. Kaiser, E.R. Encina, E.A. Coronado, G. Van Tendeloo. 3D imaging of nanomaterials by discrete tomography. *Ultramicroscopy* **109**, 730-740 (2009).

15 Fisher, S. B. On the temperature rise in electron irradiated foils. *Radiation Defects* **5**, 239-243 (1970).

16 I. Jencic, M. W. B., I.M. Robertson, M.A. Kirk. Electron-beam-induced crystallization of isolated amorphous regions in Si, Ge, GaP, and GaAs. *Journal of Applied Physics* **78**, 974-982 (1995).

17 Sigmund, P. in *Springer Series in Solid State Sciences* Vol. 151 (Springer-Verlag, 2006).

18 Egerton, R. Radiation damage in the TEM and SEM. *Micron* **35**, 399-409 (2004).

19 Hobbs, L. W. in *Introduction to Analytical Electron Microscopy* (ed J.I. Goldstein J.J. Hren, D.C. Joy) 399-445 (Plenum Press, 1987).
